# Quantitative Data Integration Analysis Method for Cross-Studies: Obstructive Sleep Apnea as an Example

**DOI:** 10.1155/2022/1977446

**Published:** 2022-06-07

**Authors:** Rong Zhou, Shengrong Zhou, Qiguang Xia, Tiejun Zhang, Guoqing Zhang

**Affiliations:** ^1^Department of Epidemiology, School of Public Health, Fudan University, Shanghai 200232, China; ^2^Key Laboratory of Public Health Safety, Fudan University, Ministry of Education, Shanghai, China; ^3^CAS Key Laboratory of Computational Biology, Bio-Med Big Data Center, Shanghai Institute of Nutrition and Health, University of Chinese Academy of Sciences, Chinese Academy of Science, Shanghai 200031, China; ^4^Human Phenome Institute, School of Life Sciences, Fudan University, Shanghai 200232, China

## Abstract

**Objective:**

In recent years, the prevalence of obstructive sleep apnea (OSA) has gradually increased. The diagnosis of this multiphenotypic disorder requires a combination of several indicators. The objective of this study was to find significant apnea monitor indicators of OSA by developing a strategy for cross-study screening and integration of quantitative data.

**Methods:**

Articles related to sleep disorders were obtained from the PubMed database. A sleep disorder dataset and an OSA dataset were manually curated from these articles. Two evaluation indexes, the indicator coverage ratio (ICR) and the study integrity ratio (SIR), were used to filter out OSA indicators from the OSA dataset and create profiles including different numbers of indicators and studies for analysis. Data were analyzed by the *meta 4.18-0 package* of R, and the *p* value and standard mean difference (SMD) values were calculated to evaluate the change of each indicator.

**Results:**

The sleep disorder dataset was constructed based on 178 studies from 119 publications, the OSA dataset was extracted from 89 studies, 284 sleep-related indicators were filtered out, and 22 profiles were constructed. Apnea hypopnea index was significantly decreased in all 22 profiles. Total sleep time (TST) (min) showed no significant differences in 21 profiles. There were significant increases in rapid eye movement (REM) (%TST) in 18 profiles, minimum arterial oxygen saturation (SaO_2_) in 9 profiles, REM duration in 3 profiles, and slow wave sleep duration (%TST) and pulse oximetry lowest point in 2 profiles. There were significant decreases in apnea index (AI) in 14 profiles; arousal index and SaO_2_ < 90 (%TST) in 8 profiles; N1 stage (%TST) in 7 profiles; and hypopnea index, N1 stage (% sleep period time (%SPT)), N2 stage (%SPT), respiratory arousal index, and respiratory disorder index in 2 profiles.

**Conclusion:**

The proposed data integration strategy successfully identified multiple significant OSA indicators.

## 1. Introduction

With the advent of medical big data, medical research began to involve collecting data through a variety of data acquisition systems and exploring disease risk factors, factors affecting treatment compliance, and disease prediction model construction methods using machine learning and deep learning technologies [1–3]. Big data enables more powerful evaluations of health care quality and efficiency, which can be used to promote care improvement [4]. Meta-analysis is extremely valuable for decision-making, because it eliminates the majority of noise caused by individual studies, making the results more convincing. Selection bias may occur if researchers do not clearly define the criteria for choosing studies from the long list of potentially suitable studies [5]. If the data collection method under the background of big data is applied and computer code is used to calculate and screen data from multiple studies, the bias of subjective data screening by reviewers may be avoided.

Obstructive sleep apnea (OSA) is the most common type of sleep-disordered breathing, according to the American Academy of Sleep Medicine classification. The assessment of OSA patients relies on polysomnography (PSG) [6], home portable sleep monitor [7], functional outcome of sleep questionnaire (FOSQ) [8], and the Epworth sleepiness scale (ESS) [9]. PSG is the gold standard for OSA diagnosis, but its widespread use is hindered by long waiting times and high costs. In addition, some patients have atypical symptoms and lack sleep medicine knowledge, leading to difficulties during the diagnosis of OSA [7]. Clinical patients need to be evaluated regularly for disease severity, but cost and time need to be considered. Comprehensive analysis of OSA study data is important for determining the best and most cost- and time-effective OSA indicators [10].

Although the apnea hypopnea index (AHI) is the main indicator for OSA, it neglects the duration of apnea and the severity of blood oxygen saturation, so more comprehensive paradigms are urgently needed [11]. In previous meta-analysis, ESS [12] and minimum arterial oxygen saturation (SaO_2_) [13] were occasionally used for complementary diagnosis alongside AHI [13–15]. Although some phenotypic information of OSA, such as daytime sleepiness and arousal threshold, has been studied, more phenotypic identification of OSA is still needed to gain a deeper understanding of the disorder [16] and uncover true associations that may be masked by unstandardized and imperfect phenotypes. Some new clinical trials suggest that focusing on other indicators may be useful for follow-up of OSA, such as pulse oximetry- (SpO_2_-) related indicators [17] and oxygen desaturation index (ODI) [10]. Therefore, the integration of common indicators reported in previous OSA studies can provide reference information for clinical studies to identify more indicators related to OSA.

To find significant indicators of OSA from multiple studies, a strategy for cross-study screening and integration of quantitative OSA data was constructed. The aim of this study was to construct an OSA dataset from clinical trials (CTs) and randomized controlled trials (RCTs), which would contain multiple indicators from multiple studies, apply two evaluation indexes to filter out OSA indicators, and determine the best indicators for OSA assessment and surveillance.

## 2. Materials and Methods

### 2.1. Literature Search and Study Selection

**Figure 1:**
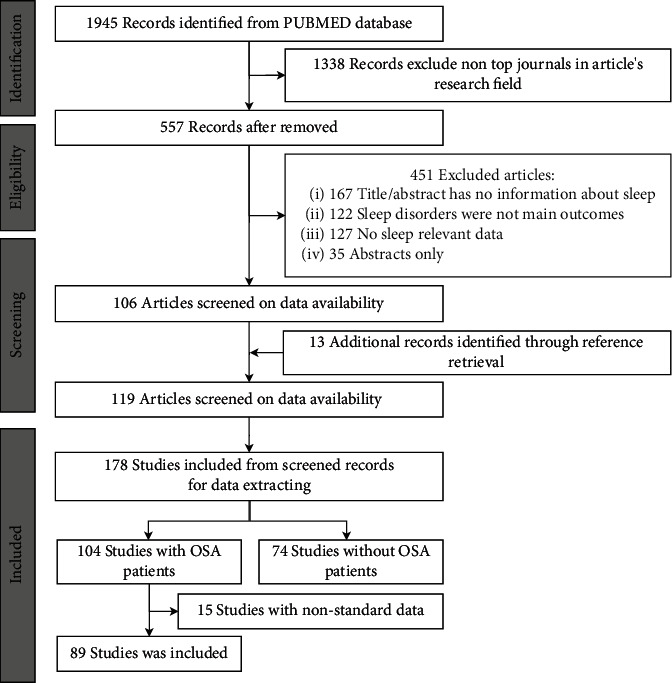
Flowchart of literature search and study selection process.

To review the previous published literature on sleep disordered breathing, sleep monitoring, and sleep-related diseases [16, 18, 19], we obtained the keywords of sleep assessment, related disease diagnosis information, and phenotypes and then integrated and summarized the information to construct the search strategy according to the Preferred Reporting Items for Systematic Reviews and Meta-Analyses (PRISMA) guidelines [20]. The PRISMA checklist is shown in Supplementary Table 1. Key search terms included Sleep, Arousals, Apnea-Hypopnea Index, Electroencephalogram, Rapid eye movement sleep behavior disorder (RBD), Sleep Structure, and non-rapid eye movement. Retrieval content was limited to title, the publication types were limited to CT and RCT, and the time cutoff was December 18, 2020. The flowchart of the literature search and study selection process is shown in Figure 1. Details of search terms are reported in Supplementary Table 2.

The initial screening of titles and abstracts of publications was based on the following two criteria. Inclusion criteria: (1) journals are within their top respective sectors; (2) sleep monitoring data were available; (3) sleep-disordered breathing was the main outcome; (4) the research subjects were human. Exclusion criteria: (1) systematic review, review, and meta-analysis; (2) the research subjects were animals; (3) only abstracts were available.

The eligible publications with full text were reviewed, and the studies were separated into two subgroups: those of OSA patients and those of non-OSA patients, and the former was selected for integrated analysis.

### 2.2. Data Extraction and Risk Assessment

To obtain comprehensive and accurate research information, the extracted contents included general publication data, study design, duration of study, demographic variables, and intervention methods; specific data extraction information is shown in Table 1.

OSA indicators with the same meaning could be denoted by different terms in various publications. To reflect this phenomenon, these different terms were retained in the OSA dataset. For example, AHI and other similar indicators were recorded dependently, such as AHI during supine sleep, AHI during nonsupine sleep, AHI during rapid eye movement (REM) stage, and AHI during nonrapid eye movement (NREM) stage. Data included baseline and follow-up or control and experimental group, and indicators were recorded as the indicator name with suffix “_Baseline” and “_Follow-up,” respectively. All studies identified by the research were independently screened for eligibility by two investigators (RZ and SZ). Data collection was conducted using Microsoft Excel (V2019, Microsoft, USA, 2019). Reviewers reviewed the risk of bias of individual studies using the Cochrane Collaboration's tool for assessing risk of bias [21] and RevMan software (version 5.3). Discordances were discussed with a third reviewer (GZ) and resolved by consensus.

### 2.3. Indicator Evaluation

To evaluate data of indicators and studies, two indexes were calculated: the coverage of each sleep monitoring indicator, denoted as the indicator coverage ratio (ICR), and the integrity of each study under the ICR threshold, denoted as the study integrity ratio (SIR). Their calculation formulas were as follows:

(1) $$\begin{array}{c} \text{ICR}=\overset{n}{\underset{i=1}{\sum }}\frac{S_{i}}{T_{s}}, \end{array}$$

(2) $$\begin{array}{c} \text{SIR}=\overset{n}{\underset{s=1}{\sum }}\frac{I_{s}}{I_{t}}, \end{array}$$

where *S*_*i*_ is the study with indicator, *n* is the total number of indicators, *i* is the identifying number of an indicator, *T*_*s*_ is the total number of studies included, *I*_*s*_ are indicators in a study under indicator coverage ratio threshold, *s* is the identifying number of a study, and *T*_*t*_ is the total number of indicators under indicator coverage ratio threshold.

ICR reflects the usage degree for each indicator in all the included studies, and SIR reflects the number of indicators in each study under different ICR thresholds. The ICR was calculated and then divided into 10 ICR thresholds (ICRTs) from 0.1 to 1.0. Then, the SIR of each study was calculated under each ICRT, and the SIR was set to 0.1–1.0 for a total of 10 SIR thresholds (SIRTs). The different ICRTs and SIRTs were combined into 22 profiles including different numbers of indicators and studies, and then, these profiles were analyzed. For the same ICRT, if the same study was filtered by different SIRTs, the study was deduplicated, and the higher SIRT was retained. Each profile was described according to the values of ICRT and SIRT, such as P1-1 for ICRT = 0.1 and SIRT = 0.1 and P7-10 for ICRT = 0.7 and SIRT = 1.0. The data screening process was performed using Python 3.9.

### 2.4. Statistical Analysis

Quantitative data integration analysis adopted the analysis principle of meta-analysis and was conducted by a package called *meta 4.18-0* (R software version 4.0.5; R Foundation for Statistical Computing, Vienna, Austria). Each indicator retained under the different combinations of ICRT and SIRT was independently analyzed, and studies with missing values were excluded from each analysis. A fixed effects model was used for analysis. A *χ*^2^ test and an *I*^2^ test were used to test the heterogeneity of the included studies. If *p* < 0.05 and *I*^2^ > 50% were shown, the random effect model was employed. A *p* value less than 0.05 was considered to indicate statistical significance (*α* = 0.05). The SMD value indicated a change of the quantitative indicators with a significant *p* value. Publication bias was evaluated through a funnel plot.

## 3. Results

### 3.1. Identification and Selection of Studies

The literature search yielded 1945 nonduplicate studies from the PubMed database through the retrieval strategy. After excluding 1388 studies that were in the nontop journals in their research field or were systematic reviews, reviews, and meta-analyses, 557 publications were further reviewed. Among them, 167 had titles and abstracts that did not provide any information about sleep, 122 were non-sleep-related studies, 127 had data that could not be obtained, and 35 did not have full English text, so they were all excluded, and the remaining 106 studies were included for further review. In addition, 13 studies from other publications, which met the inclusion criteria, were included for review. Then, data were obtained from 178 studies from 119 publications, including 104 studies whose subjects were OSA. Eighty-nine studies with mean and standard deviation data were further selected for the final analysis. All collected research information and data can be seen in Supplementary Table 3.

### 3.2. Study Characteristics and Risk of Bias Assessment

**Figure 2:**
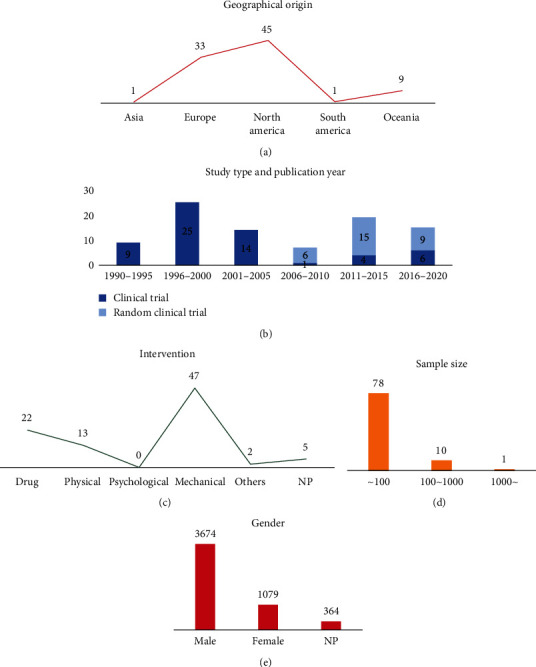
Overview of trends in studies for OSA patients.

**Figure 3:**
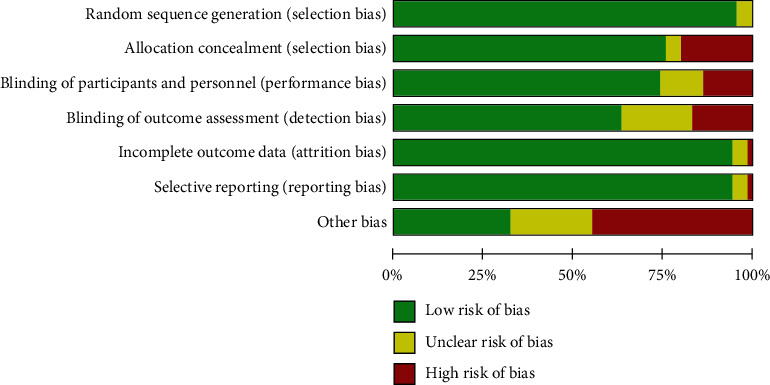
Risk of bias item presented as percentages across all included studies.

Among the 89 included studies, the most common study regions were Europe (*n* = 33) and North America (*n* = 45). All studies spanned a period between 1990 and 2020. The most common sample size was less than 100 (*n* = 78). A total of 5117 OSA patients were included in this analysis, including 3674 males and 1079 females. A total of 30 RCT studies and 59 CT studies were identified (Figure 2). All studies were assessed using the Cochrane Collaboration's tool for assessing risk of bias, and most of the studies had low risk of bias and mentioned randomization. Bias in other studies was mainly derived from the small sample size of the study. The details of risk of bias assessment are presented in Figure 3.

### 3.3. Indicator Coverage Ratio and Study Integrity Ratio

**Figure 4:**
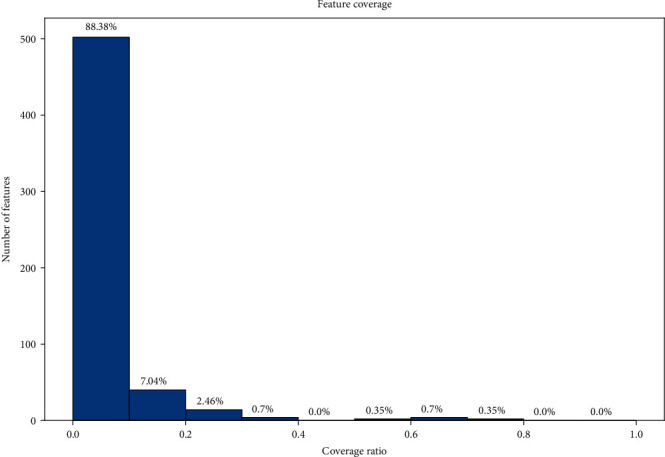
ICR of 284 sleep monitor indicators.

A total of 284 sleep-related monitor indicators were identified from the 89 studies.  Figure 4 shows the percentage of indicators under the ICRT. There were 251 (88.38%) indicators with coverage between 0.0 and 0.1, 20 (7.04%) indicators with coverage between 0.1 and 0.2, 7 (2.46%) indicators with coverage between 0.2 and 0.3, 2 (0.7%) indicators with coverage between 0.3 and 0.4, no (0.0%) indicators with coverage between 0.4 and 0.5, 1 (0.35%) indicator with coverage between 0.5 and 0.6, 2 (0.7%) indicators with coverage between 0.6 and 0.7, 1 (0.35%) indicator with coverage between 0.7 and 0.8, and no (0.0%) indicators with coverage threshold greater than 0.8. The SIR was calculated for the ICRT. Specific information on the indicators and studies selected in different profiles is shown in Supplementary Table 4.

### 3.4. Integrated Analysis Results

Twenty-two combination profiles of ICRT and SIRT were selected for analysis. When the ICRT was 0.1 and the SIRT was 0.1 (P1-1), 33 sleep indicators were retained for analysis, and a total of 22 indicators showed statistical significance. When the ICRT was 0.7 and the SIRT was 1.0 (P7-10), only one indicator was included, and the indicator had significant change.

There were significant reductions in hypopnea index (HI), N1 stage (% sleep period time (%SPT)), N2 stage (%SPT), respiratory arousal index, and respiratory disorder index (RDI) in two profiles; N1 stage (% total sleep time (%TST)) in 7 profiles; arousal index and SaO_2_ < 90 (%TST) in 8 profiles; and apnea index (AI) in 14 profiles. There were significant increases in slow wave sleep (SWS) duration (%TST) and SpO_2_ lowest point in 2 profiles, REM duration in 3 profiles, minimum SaO_2_ in 9 profiles, and REM (%TST) in 18 profiles. The TST (min), N1+N2 (%TST), N2 (%TST), N3 (%TST), N4 (%TST), ODI, NREM sleep duration, REM latency, and sleep latency did not change significantly.

**Figure 5:**
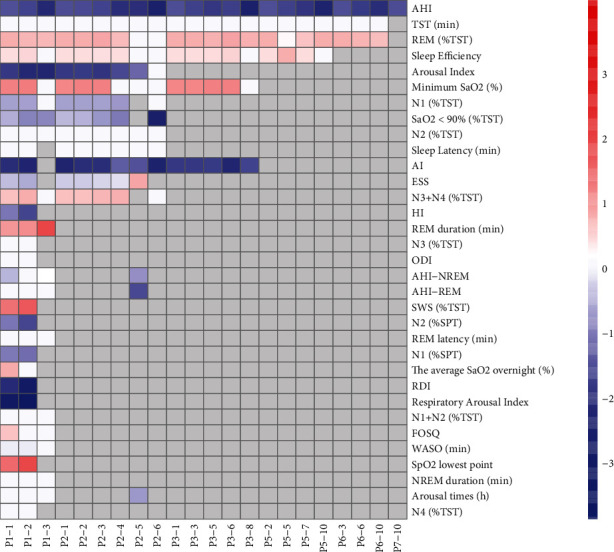
ICR and SIR threshold analysis results. AHI: apnea hypopnea index; AHI-NREM: apnea hypopnea index in nonrapid eye movement sleep; AHI-REM: apnea hypopnea index in rapid eye movement sleep; AI: apnea index; ESS: Epworth sleepiness score; FOSQ: functional outcomes of sleep questionnaire; HI: hypopnea index; SPT: sleep period time; TST: total sleep time; NREM: nonrapid eye movement; REM: rapid eye movement; ODI: oxygen desaturation index; REM (%TST): the percentage of rapid eye movement of total sleep time; RDI: respiratory disorder index; SaO_2_ < 90% (%TST): percentage of total sleep time with SaO_2_ < 90%; SWS: slow wave sleep; WASO: wake after sleep onset. ^∗^The white block is *p* < 0.05 of the indicator analysis; we consider that there is no significant change in SMD and assign the SMD value to 0.

**Figure 6:**
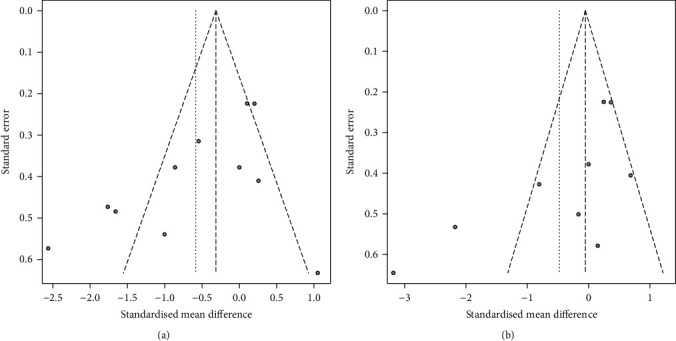
Funnel plots of two dependent analysis. (a) Funnel plot of AHI-REM of 11 studies in profile P1-1. (b) Funnel plot of AHI-REM of 9 studies in profile P1-2.

Among all 22 profiles, AHI was the most common OSA indicator, and it exhibited a significant decrease in all 22 profiles. REM (%TST) and TST were the next most common, as they were included in 21 profiles. As previously mentioned, REM (%TST) increased significantly in 18 profiles, whereas TST (min) did not significantly change in any profiles (Figure 5). Supplementary Table 5 presents additional details of each profile result. The publication bias of the apnea hypopnea index in rapid eye movement sleep (AHI-REM) of profile P1-1 and profile P1-2 was evaluated, and no significant bias was found (Figure 6).

## 4. Discussion

In the process of monitoring the OSA dataset and filtering out OSA indicators, two indexes were adopted: ICR and SIR, which are two new evaluation methods. ICR described the overall usage of each indicator in disease monitoring studies, and SIR reflected the specific usage situation of each indicator in disease monitoring studies. ICRT and SIRT were applied to objectively filter indicators and studies for analysis. The larger the ICRT, the more common the monitoring indicator was in the included OSA-related studies, and the larger the SIRT, the greater the proportion of common indicators in each study. The result in Figure 4 shows that the coverage of most indicators was less than 0.1, and less than nine studies on these OSA-related indicators were included in the detection analysis. These indicators included SWS, supine duration, snoring duration [22], time to wake up after falling asleep [23], and REM activity density [24]. SWS was defined as stages 3 and 4 non-REM sleep [23], and instead of SWS, two indicators, N3 and N4, were recorded in some studies [25]. REM activity density has been used as an indicator to evaluate REM frequency [26], but it was not used in any of the 89 OSA studies included in our study, possibly because the change in REM density was caused by the change in REM time [27], and therefore, the REM time and REM latency were more often used for REM assessment.

The results of studies with different profiles show commonalities. AHI exhibited a decrease in all profiles, which is consistent with previous studies [28, 29]. AHI has been acknowledged as a robust endpoint for the diagnosis of OSA and the assessment of treatment efficacy and is used worldwide [30, 31]. In addition to AHI, which is defined as the average number of apnea plus hypopnea episodes during sleep, we need more indicators to better characterize OSA [32] and fully recognize changes in OSA across interventions or populations. It has been shown that reducing hypoventilation and apnea indices can significantly alleviate partial and complete obstruction in patients with mild OSA [33]. The results of our analysis showed a significant decrease in AI (13 profiles) and HI (two profiles) associated with AHI, consistent with the changes in AHI. Physicochemical studies of AHI would likely obtain relevant information enabling the discovery of more indicators.

TST showed no significant differences in 21 profiles, which was consistent with some studies [34–36]. Eighteen of 21 profiles including REM (%TST) showed significantly decreased REM. We could infer that the change of REM (%TST) was caused by the change of REM duration. Limited REM duration is thought to be one of the causes of OSA. OSA patients generally have worse REM sleep because of the degree of desaturation and duration of apnea [37], and REM represents the physical neurophysiological state and the control of ventilation of the upper airway [38]. Ventilation control is more stable in OSA patients during REM than during NREM [39], so with the increase of REM duration, the severity of disease may be relieved. REM duration is an important indicator for OSA assessment.

Effective surveillance indicators can facilitate the development of preventive strategy measures for public health [40]. The innovation of this study is the method of using two indicators to integrate data across studies, which is a new technique for objectively mining effective surveillance indicators for diseases. In addition, the results of our analysis are informative for the design of epidemiological studies and clinical trials.

One limitation of this study was that the included research data had great differences in decade, region, intervention method, gender ratio, and sample size, but no profile analysis [41], sensitivity analysis [42], or other such analysis was conducted to explain the sources of data heterogeneity. Besides, it is necessary to conduct subsequent clinical studies to further verify our results.

## 5. Conclusion

The results of this study show the feasibility and effectiveness of the data acquisition and screening strategy. We provide ICR and SIR for calculating and selecting data from multiple studies with different indicators, which can also demonstrate the usage and significance of OSA indicators. Among the 22 ICRT and SIRT combination profiles, 33 indicators were distributed across all profiles, and 15 indicators, including AHI, exhibited significant changes. In addition to AHI, other indicators for OSA monitoring and assessment merit further study. Since this study proposed a new data collection and screening strategy, more analytical and clinical studies are needed to further validate the results.

## Figures and Tables

**Table 1:** Extracted relevant data.

Extracted information	Details
General publication data	DOI
	First author
	Journal and year of publication
	Geographical origin
Study design	Randomized controlled trial (RCT)
	Clinical trial (CT)
Duration of study	Time interval
	Time follow-up
Demographic variables	Sample size of case and control profile
	Age
	Number of males and females
Intervention methods	Mechanical intervention (such as CPAP)
	Physical intervention (such as tonsillectomy and exercise therapy)
	Drug intervention (such as eszopiclone)
	Psychological intervention (such as continuous antidepressant treatment)
	Other interventions (such as low calorie intake strategies and normal lifestyle)

## Data Availability

The data used to support the findings of this study are included within the supplementary information files, which have been deposited in https://figshare.com ([DOI: 10.6084/m9.figshare.19179158]).

## Supplementary Materials

Supplementary Materials The Supplementary Material for this article: Supplementary Table 1: PRISMA 2009 checklist. Supplementary Table 2: search strategies. Supplementary Table 3: research information details and indicator dataset. Supplementary Table 4: specific information of indicators and studies under different profile. Supplementary Table 5: analysis result data.

## References

[1] Ngiam K. Y., Khor I. W. (2019). Big data and machine learning algorithms for health-care delivery. *The Lancet Oncology.*.

[2] Malhotra A., Morrell M. J., Eastwood P. R. (2018). Update in respiratory sleep disorders: epilogue to a modern review series. *Respirology (Carlton, Vic.)*.

[3] Adams J. U. (2020). Big hopes for big data. *Nature medicine*.

[4] Price W. N., Cohen I. G. (2019). Privacy in the age of medical big data. *Nature medicine*.

[5] Silva-Fernández L., Carmona L. (2019). Meta-analysis in the era of big data. *Clinical rheumatology.*.

[6] Engstrøm M., Rugland E., Heier M. S. (2013). Polysomnography (PSG) for studying sleep disorders. *Tidsskrift for den Norske laegeforening: tidsskrift for praktisk medicin, ny raekke.*.

[7] Aielo A. N., Santos R. B., Silva W. A. (2019). Pragmatic validation of home portable sleep monitor for diagnosing obstructive sleep apnea in a non-referred population: the ELSA-Brasil study. *Sleep science*.

[8] Weaver T. E., Laizner A. M., Evans L. K. (1997). An instrument to measure functional status outcomes for disorders of excessive sleepiness. *Sleep*.

[9] Johns M. W. (1991). A new method for measuring daytime sleepiness: the Epworth sleepiness scale. *Sleep*.

[10] Varghese L., Rebekah G., Priya N., Oliver R. K. A. (2022). Oxygen desaturation index as alternative parameter in screening patients with severe obstructive sleep apnea. *Sleep science*.

[11] Leppänen T., Kulkas A., Töyräs J. (2019). The hypoxic burden: also known as the desaturation severity parameter. *European heart journal.*.

[12] Javaheri S., Martinez-Garcia M. A., Campos-Rodriguez F., Muriel A., Peker Y. (2020). Continuous positive airway pressure adherence for prevention of major adverse cerebrovascular and cardiovascular events in obstructive sleep apnea. *American journal of respiratory and critical care medicine.*.

[13] Wang L., Xu J., Zhan Y., Pei J. (2020). Acupuncture for obstructive sleep apnea (OSA) in adults: a systematic review and meta-analysis. *Bio Med research international*.

[14] Lee C. H., Hsu W. C., Ko J. Y., Yeh T. H., Lin M. T., Kang K. T. (2020). Adenotonsillectomy for the treatment of obstructive sleep apnea in children with Prader-Willi syndrome: a meta-analysis. *Otolaryngology--head and neck surgery: official journal of American Academy of Otolaryngology-Head and Neck Surgery.*.

[15] Clements A. C., Dai X., Walsh J. M. (2021). Outcomes of adenotonsillectomy for obstructive sleep apnea in Prader-Willi syndrome: systematic review and meta-analysis. *The Laryngoscope.*.

[16] Zinchuk A. V., Gentry M. J., Concato J., Yaggi H. K. (2017). Phenotypes in obstructive sleep apnea: a definition, examples and evolution of approaches. *Sleep medicine reviews.*.

[17] Nigro C. A., Castaño G., Bledel I., Colombi A., Zicari M. C. (2021). A novel, simple, and accurate pulse oximetry indicator for screening adult obstructive sleep apnea. *Sleep and Breathing*.

[18] Santamaria J., Iranzo A. (2014). Sleep disorders matter in neurology. *The Lancet Neurology.*.

[19] Veasey S. C., Rosen I. M. (2019). Obstructive sleep apnea in adults. *The New England journal of medicine.*.

[20] Moher D., Shamseer L., Clarke M. (2015). Preferred reporting items for systematic review and meta-analysis protocols (PRISMA-P) 2015 statement. *Systematic reviews*.

[21] Higgins J. P. T., Altman D. G., Gøtzsche P. C. (2011). The Cochrane Collaboration’s tool for assessing risk of bias in randomised trials. *BMJ*.

[22] Guerrero A., Embid C., Isetta V. (2014). Management of sleep apnea without high pretest probability or with comorbidities by three nights of portable sleep monitoring. *Sleep*.

[23] Lapierre O., Montpiaisir J., Lamarre M., Bedard M. A. (1990). The effect of gamma-hydroxybutyrate on nocturnal and diurnal sleep of normal subjects: further considerations on REM sleep-triggering mechanisms. *Sleep*.

[24] Hornung O. P., Regen F., Schredl M., Heuser I., Danker-Hopfe H. (2006). Manipulating REM sleep in older adults by selective REM sleep deprivation and physiological as well as pharmacological REM sleep augmentation methods. *Experimental neurology.*.

[25] Kluge M., Gazea M., Schüssler P. (2010). Ghrelin increases slow wave sleep and stage 2 sleep and decreases stage 1 sleep and REM sleep in elderly men but does not affect sleep in elderly women. *Psychoneuroendocrinology*.

[26] Steiger A., Pawlowski M. (2019). Depression and sleep. *International journal of molecular sciences*.

[27] Antonioli M., Solano L., Torre A., Violani C., Costa M., Bertini M. (1981). Independence of REM density from other REM sleep parameters before and after REM deprivation. *Sleep*.

[28] Liming B. J., Ryan M., Mack D., Ahmad I., Camacho M. (2019). Montelukast and nasal corticosteroids to treat pediatric obstructive sleep apnea: a systematic review and meta-analysis. *Otolaryngology--head and neck surgery: official journal of American Academy of Otolaryngology-Head and Neck Surgery.*.

[29] Andrade R. G. S., Viana F. M., Nascimento J. A. (2018). Nasal vs oronasal CPAP for OSA treatment: a meta-analysis. *Chest*.

[30] Cielo C. M., Tapia I. E. (2019). Diving deeper: rethinking AHI as the primary measure of OSA severity. *Journal of clinical sleep medicine: JCSM: official publication of the American Academy of Sleep Medicine.*.

[31] Cooksey J. A., Balachandran J. S. (2016). Portable monitoring for the diagnosis of OSA. *Chest*.

[32] Azarbarzin A., Sands S. A., White D. P., Redline S., Wellman A. (2019). The hypoxic burden: a novel sleep apnoea severity metric and a predictor of cardiovascular mortality-reply to ‘The hypoxic burden: also known as the desaturation severity parameter’. *European heart journal.*.

[33] Tuomilehto H. P., Seppä J. M., Partinen M. M. (2009). Lifestyle intervention with weight reduction. *American journal of respiratory and critical care medicine.*.

[34] Pitsis A. J., Darendeliler M. A., Gotsopoulos H., Petocz P., Cistulli P. A. (2002). Effect of vertical dimension on efficacy of oral appliance therapy in obstructive sleep apnea. *American journal of respiratory and critical care medicine.*.

[35] Gagnadoux F., Pépin J. L., Vielle B. (2017). Impact of mandibular advancement therapy on endothelial function in severe obstructive sleep apnea. *American journal of respiratory and critical care medicine.*.

[36] Smales E. T., Edwards B. A., Deyoung P. N. (2015). Trazodone effects on obstructive sleep apnea and non-REM arousal threshold. *Annals of the American Thoracic Society.*.

[37] Siddiqui F., Walters A. S., Goldstein D., Lahey M., Desai H. (2006). Half of patients with obstructive sleep apnea have a higher NREM AHI than REM AHI. *Sleep medicine.*.

[38] Joosten S. A., Hamza K., Sands S., Turton A., Berger P., Hamilton G. (2012). Phenotypes of patients with mild to moderate obstructive sleep apnoea as confirmed by cluster analysis. *Respirology*.

[39] Landry S. A., Andara C., Terrill P. I. (2018). Ventilatory control sensitivity in patients with obstructive sleep apnea is sleep stage dependent. *Sleep*.

[40] Jarris P. E. (2013). Obesity as disease. *Journal of public health management and practice: JPHMP.*.

[41] Berry R. B., Kryger M. H., Massie C. A. (2011). A novel nasal expiratory positive airway pressure (EPAP) device for the treatment of obstructive sleep apnea: a randomized controlled trial. *Sleep*.

[42] Tanner-Smith E. E., Grant S. (2018). Meta-analysis of complex interventions. *Annual review of public health.*.

