# Supplementary material for: Quantitative Data Integration Analysis Method for Cross-Studies: Obstructive Sleep Apnea as an Example
Source: Comput Math Methods Med. 2022 Jun 7;2022:1977446. doi: 10.1155/2022/1977446 (PMC9197656; doi:10.1155/2022/1977446)
Supplement: Supplementary Materials — The Supplementary Material for this article: Supplementary Table 1: PRISMA 2009 checklist. Supplementary Table 2: search strategies. Supplementary Table 3: research information details and indicator dataset. Supplementary Table 4: specific information of indicators and studies under different profile. Supplementary Table 5: analysis result data. [file 1977446.f1.zip › Supplementary Table2.docx]

**Supplementary Table 1**

| **Search Strategy for PubMed** | |
| --- | --- |
| **Key words** | (Sleep[Title] OR Sleeping[Title]OR dormant[Title] ) AND ((ECG[Title] OR Electrocardiogram[Title]) OR (EEG[Title] OR Electroencephalogram[Title]) OR (EOG[Title] OR Electro oculogram[Title]) OR (Thoracic movement[Title] OR Abdominal movement[Title]) OR (Sleep stage[Title] OR Sleep structure[Title]) OR (NREM[Title] OR non-rapid eye movement[Title]) OR (REM[Title] OR rapid eye movement[Title]) OR (Pittsburgh questionnaire[Title] OR Post-sleep questionnaire[Title]) OR (Oxygen saturation[Title] OR Oxygen desaturation[Title]) OR Body position[Title]OR Sleep Latency[Title] OR (AHI[Title] OR Apnea–Hypopnea Index[Title]) OR (LM[Title] OR Limb movement[Title])OR( Arousals[Title] OR Apnea[Title] OR Snoring[Title] OR Heart rate[Title])OR ( sleep Circadian Rhythm[Title] OR Hypopnea[Title]OR (bradycardia[Title] OR tachycardia[Title] OR Cardiac asystole[Title]) OR Atrial fibrillation[Title] OR (rhythmic movement disorder[Title] OR rapid eye movement sleep behavior disorder[Title] OR RMD[Title]) OR TMA[Title] OR SMA[Title]) OR (sleep wake disorder[Title]) OR (sleep disorder[Title])) |
| **Publication period** | (" 1000/1/1" : "2020/12/18"[PDat]) |
| **Search filters** | Filters: Clinical Trial, Randomized Controlled Trial |
